# Complete Genome Sequence of the Ice-Nucleation-Active Pseudomonas syringae Strain MUP20, Isolated from Frost-Damaged Wheat (Triticum aestivum cv. Scepter) in Western Australia

**DOI:** 10.1128/mra.01275-22

**Published:** 2023-02-13

**Authors:** Hussain Alattas, Julie Ardley, Rebecca Swift, Sarah Jackson, Ben Biddulph, Amanuel Bekuma, Jaco Zandberg, Sam Abraham, Ravi Tiwari, Wayne Reeve

**Affiliations:** a Centre for Crop and Food Innovation, Food Futures Institute, Murdoch University, Murdoch, Western Australia, Australia; b School of Molecular and Life Sciences, Faculty of Science and Engineering, Curtin University, Perth, Western Australia, Australia; c Department of Primary Industries and Regional Development, Western Australia, Perth, Western Australia, Australia; d Antimicrobial Resistance and Infectious Diseases Laboratory, Harry Butler Institute, Murdoch University, Murdoch, Western Australia, Australia; University of Arizona

## Abstract

Pseudomonas syringae MUP20 was isolated from Western Australian frost-damaged wheat. The MUP20 complete genome contained a 6,045,198-bp single circular chromosome with a GC content of 59.03%. IMG/M genome annotation identified 5,245 protein-coding genes, 1 of which encoded an ice nucleation protein containing 20 occurrences of a highly repetitive PF00818 domain.

## ANNOUNCEMENT

The Pseudomonas syringae species complex (1, 2) is divided into 13 distinct phylogroups, with many strains containing ice-nucleation proteins that contribute to frost damage in crops (3, 4). As part of an ongoing study, we have been identifying bacteria that dominate frost-damaged crops across Australia. Here, we report the complete genome sequence of a P. syringae isolate designated MUP20 (= WAC15075), which was isolated (5) from mid-April-sown wheat (Triticum aestivum cv. Scepter) that had been frost damaged at the reproductive stage and was collected from a frost trial site in Wickepin, Western Australia, Australia, in 2018. Further details of the trial can be found in previous research (6).

A pure culture of this strain was grown in lysogeny broth (LB) and cryopreserved in 15% glycerol at −80°C. High-molecular-weight genomic DNA was isolated from a logarithmic-phase culture as described previously (7), and the same DNA preparation was sequenced using both Illumina and Oxford Nanopore Technologies (ONT) platforms. ONT libraries were prepared according to the ONT 1D ligation library preparation protocol (SQK-LSK109) and sequenced with a FLO-MIN-106D flow cell (R9.4.1) on a MinION platform. Guppy v3.2.6 was used for base calling with a read-pass-filter quality score cutoff value of 7. A total of 33,311 ONT reads were generated, producing a total of 344,055,820 bp with ~60× coverage and a read *N*_50_ value of 10,329 bp.

An Illumina library was prepared with the NuGEN Celero DNA-sequencing library preparation kit, following the manufacturer’s protocol. The library was sequenced on an Illumina NextSeq platform using a midoutput 2 × 150-bp kit. This workflow generated a total of 1,242,700 paired reads, producing a total of 178,977,604 bp with ~30× coverage of the genome.

**FIG 1:**
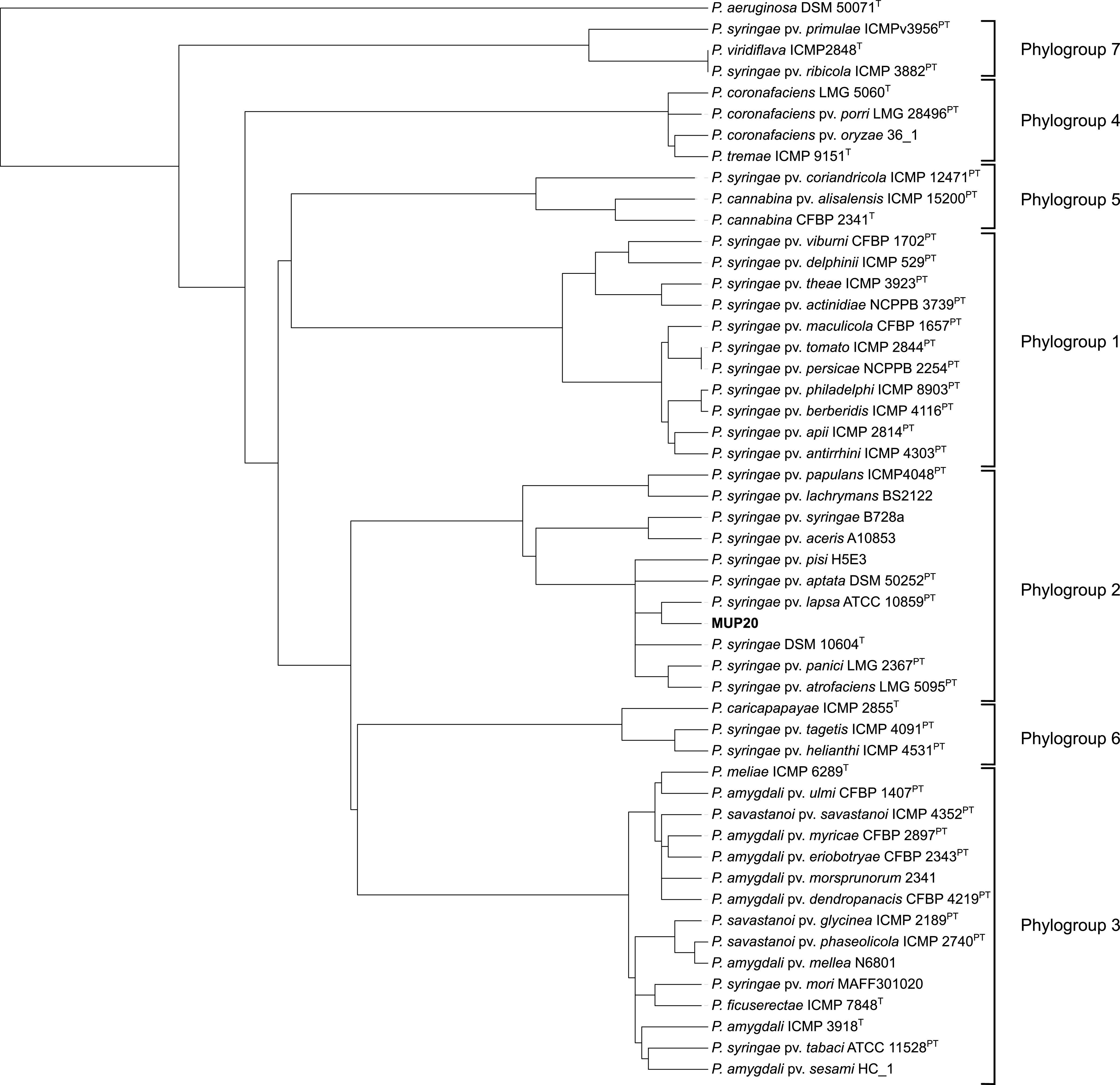
DendroUPGMA-generated tree displaying the relatedness of Pseudomonas genomes based on ANIb values. The ANIb values were generated using FastANI (12) and imported into DendroUPGMA, and the tree was constructed using a similarity matrix within the algorithm (13). The DendroUPGMA-generated tree was exported into Interactive Tree Of Life (iTOL) (14) for visualization. The superscript T indicates type strains, and the superscript PT indicates pathotype and type strains. Pseudomonas aeruginosa DSM 50071^T^ was used as an outgroup.

ONT long reads were assembled using the Flye v2.9 assembler (8) with default parameters, with 10 iterations. Short reads were mapped to the long-read assembly using minimap2 (9) to correct ONT-generated sequencing errors. The final genome contained a single circular chromosome (6,045,198 bp), with a GC content of 59.03%. The resulting chromosome was manually oriented using Geneious Prime (10) to position the *dnaA* gene at the first position in the sequence. A quantitative assessment of the genome assembly was performed using BUSCO v5.3.2 (11), which provided a completeness score of 99.6%. A comparison of average nucleotide identity using BLAST (ANIb) (12–14) values for Pseudomonas genomes revealed that MUP20 is a P. syringae strain (97.9% identity of the genome to the P. syringae type strain DSM 10604 (GenBank accession number NZ_JALK00000000.1)) that belongs in phylogroup 2 (Fig. 1).

Gene calling and annotation of the generated chromosomal sequence were performed using the IMG/M annotation pipeline (IMGAP) v5.1.8 (15) and the NCBI Prokaryotic Genome Annotation Pipeline (PGAP) (16). A genome annotation comparison is summarized in Table 1.

**TABLE 1:** General features of the genome using different annotation pipelines

Feature	Data from:	
	GenBank annotation	IMG/M annotation
Total no. of genes	5,272	5,398
No. of protein-coding genes	5,101	5,245
No. of rRNA operons	5	5
5S	6	6
16S	5	5
23S	5	5
No. of tRNA genes	63	65
No. of other RNA genes	4	4
Locus tag prefix	OQB65_	Ga0569611_01_

Of particular interest was the identification of a gene (locus tag Ga0569611_01_1668916_1672938) encoding an ice-nucleating protein that contained 20 repeats of the ice-nucleation domain PF00818 (17).

### Data availability.

The whole-genome sequence for P. syringae MUP20 has been deposited in the publicly facing Genomes OnLine Database (GOLD) v8 (18) (project identification number Gp0618315), GenBank database (accession number CP110808), and IMG/M database (genome identification number 2974440541). The raw sequencing reads have been deposited in GenBank under the accession numbers SRR22226935 and SRR22252531 for long reads and short reads, respectively.
